# Successful treatment of lathosterolosis: A rare defect in cholesterol biosynthesis—A case report and review of literature

**DOI:** 10.1002/jmd2.12158

**Published:** 2020-08-18

**Authors:** Joy Yaplito‐Lee, Gautham Pai, Winita Hardikar, Kai M. Hong, James Pitt, Justine Marum, David J. Amor

**Affiliations:** ^1^ Department of Paediatrics University of Melbourne Melbourne Victoria Australia; ^2^ Department of Gastroenterology and Clinical Nutrition Royal Children's Hospital Melbourne Victoria Australia; ^3^ Murdoch Children's Research Institute University of Melbourne Melbourne Victoria Australia; ^4^ Victorian Clinical Genetics Services Murdoch Children's Research Institute Melbourne Victoria Australia; ^5^ Department of Metabolic Medicine Royal Children's Hospital Melbourne Victoria Australia

**Keywords:** cataract, disorder of cholesterol biosynthesis, exome sequencing, fibroscan, lathosterolosis, plasma sterol assay

## Abstract

Lathosterolosis is a rare autosomal recessive disorder of cholesterol biosynthesis. It is caused by defects in the SC5D (sterol C5‐desaturase) gene which encodes for the 3‐beta‐hydroxysteroid‐delta‐5‐desaturase (also called sterol‐C5‐desaturase or lathosterol dehydrogenase). Only six cases have been described in the literature, but it is possible that a number of patients with milder forms of the condition might have been missed. Lathosterolosis manifests as microcephaly, bilateral cataracts, dysmorphism, limb anomalies, and developmental delay/intellectual disability. Liver involvement is variable and can range from normal liver function tests to portal fibrosis and cirrhosis. Diagnosis is made by demonstration of specific mutations in the *SC5D* gene and by plasma sterol analysis to confirm elevated lathosterol levels. In this report, we describe a girl with transaminitis in association with developmental delay/intellectual disability, facial dysmorphism, limb anomalies, and bilateral cataracts. Fibroscan showed severe liver fibrosis. Plasma sterol analysis and exome sequencing confirmed the diagnosis of lathosterolosis. Simvastatin treatment resulted in lowering of plasma lathosterol levels, improvement in transaminitis, and liver fibrosis grade, suggesting that children with this condition should be actively treated in order to prevent progression of liver disease.

**SYNOPSIS**

If the presence of distinctive facial features and limb anomalies raises the suspicion of cholesterol synthesis defect, testing of full sterol profile is of utmost importance since an early intervention improving the metabolic imbalance may have an impact on the patient's prognosis. Simvastatin, an inhibitor of the proximal cholesterol biosynthesis, may be useful in decreasing the level of sterol precursors in disorders of cholesterol biosynthesis.

## 1 INTRODUCTION

Cholesterol is an essential metabolite of cell membrane structure and myelin and is the precursor of steroid hormones, bile acids and other oxysterols which play a crucial role in biological functions throughout the body. Defects in several stages of cholesterol biosynthesis have been identified and their phenotypes described.^1^ It is still unclear whether the clinical manifestations are caused by reduction in cholesterol levels or an accumulation of excess precursors upstream in the cholesterol synthesis pathway. Lathosterolosis is a rare disorder of cholesterol biosynthesis. It is an autosomal recessive disorder caused by defects in the *SC5D* (sterol C5‐desaturase) gene which encodes for the enzyme 3‐beta‐hydroxysteroid‐delta‐5‐desaturase (also called sterol‐C5‐desaturase or lathosterol dehydrogenase). The enzyme 3‐beta‐hydroxysteroid‐delta‐5‐desaturase catalyses the conversion of lathosterol to 7‐dehydrocholesterol, the penultimate enzymatic reaction in the cholesterol synthesis pathway. Lathosterolosis manifests as microcephaly, bilateral cataracts, dysmorphism, limb anomalies, developmental delay/intellectual disability, and liver involvement. Only six cases have been described in the literature thus far.^2^, ^3^, ^4^, ^5^, ^6^, ^7^ In this report, we describe a girl with transaminitis in association with developmental delay/intellectual disability, subtle facial dysmorphism, limb anomalies, and bilateral cataracts. Simvastatin treatment resulted in lowering of plasma lathosterol levels, improvement in transaminitis, and liver fibrosis score as evidenced by fibroscan.

## 2 CASE REPORT

**FIGURE 1:**
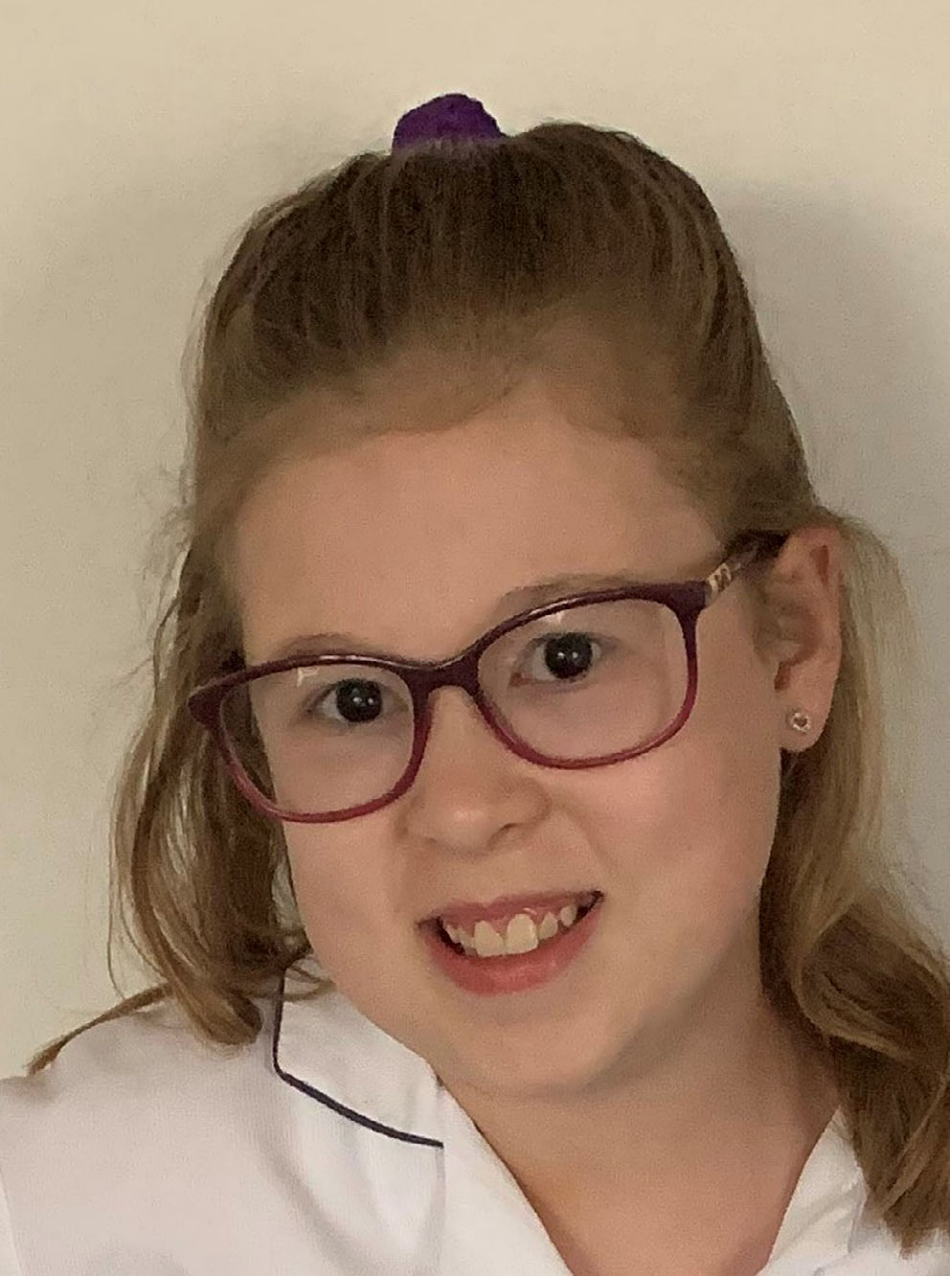
Facial view of patient

The female child was the first child of healthy unrelated parents. She was born at term following a pregnancy that was complicated by an increased nuchal fold (8.3 mm) and detection of horseshoe kidney at 20 week ultrasound. Amniocentesis was performed and karyotype showed a paternally inherited paracentric inversion, *inv* (9)*(q13q21.12)*, that was assessed as likely benign. She had a birth weight of 2680 g (10th centile), and perinatal history was unremarkable. She demonstrated early motor and language skills delay. She sat unassisted at 9 months of age, crawled at 10 months, and walked at age 22 months. She spoke her first words at 18 months of age and two word utterances at 2.5 years of age. By 8 years of age, she was behind her peers in all academic areas and attending mainstream school with an integration aid. A formal assessment using Wechsler Intelligence Scale for Children—Fifth Edition (WISC‐V) showed that her general intellectual functioning was in the extremely low range (first percentile). At 5 years of age, she was noted to have bilateral cataracts and subsequently underwent capsulotomies. Examination findings at age 7 years included height and weight on the 10th centile and head circumference 1.8 cm below the second centile. She had subtle facial dysmorphism including mild hypotelorism, epicanthic folds and retrognathia (Figure 1). She had partial 2 to 3 toe syndactyly, fifth finger clinodactyly, and dry skin. She did not have jaundice, pruritus or hepatosplenomegaly.

Liver function was first assessed at age 7 years and showed alanine aminotransferase (ALT) 321 IU/L (5‐30), gamma‐glutamyl transferase (GGT) 317 U/L (5‐20), and alkaline phosphatase (ALP) 436 U/L (120‐350). Serum bilirubin, albumin, urea, creatinine and coagulation profile were within normal range. A full blood count was essentially normal except for large platelets as evidenced by increased mean platelet volume (MPV) values ranging from 10.9 to 11.3 fL (normal 7.5‐10.4 fL). Chromosome microarray, fragile X test, urine amino and organic acids and galactoscreen were normal. Abdominal ultrasound showed a liver and spleen of normal size and echotexture, and a horseshoe kidney. Investigations for infection‐related hepatitis, autoimmune liver disease, alpha‐1‐antitrypsin deficiency, Wilson disease and coeliac disease were negative. Fasting total bile acid levels were performed using a recombinant 3‐alpha hydroxysteroid dehydrogenase enzymatic kit that measures 3‐alpha hydroxyl steroids only and results showed slightly elevated level at 10 μmol/L (normal range 1‐9 μmol/L). Plasma very long chain fatty acid (VLCFA) and phytanic acid levels were normal.

She underwent liver biopsy at 8 years of age which demonstrated a mild portal and lobular hepatitis with preserved lobular architecture. Portal tracts showed mild focal fibrosis with occasional bridging fibrosis. There were no features of autoimmune hepatitis or metabolic liver disease. Electron microscopy revealed prominent and dilated endoplasmic reticulum containing finely flocculent content. The ultrastructure of mitochondria, lysosomes, peroxisomes and bile canaliculi appeared normal.

Exome sequencing of the patient was performed. A missense variant, NM_006918.4 (SC5D):c.212A>T;p.(Asn71Ile) and a nonsense variant, NM_006918.4(SC5D):c.214C>T; p.(Gln72*) were identified in exon 3 of the *SC5D* gene. Based on ACMG variant classification guidelines both variants were classified as likely pathogenic. The presence of these two variants *in trans* confirmed a compound heterozygous mode of inheritance, consistent with lathosterolosis.

Subsequent analysis of plasma sterol levels revealed abnormally high lathosterol level (54 μmol/L, normal < 10), normal 7‐dehydrocholesterol, total cholesterol, and elevated 8(9)‐cholestenol levels, confirming the diagnosis of lathosterolosis.

Her fibroscan results suggested severe fibrosis (median reading 15.6 kPa, normal levels in children 2.5‐8.5 kPa). 25‐OH vitamin D levels were 65 to 80 nmol/L (RR > 50). Bone mineral density was low when compared to age matched controls, but it was in the lower 25% of normal with height adjustment.

**FIGURE 2:**
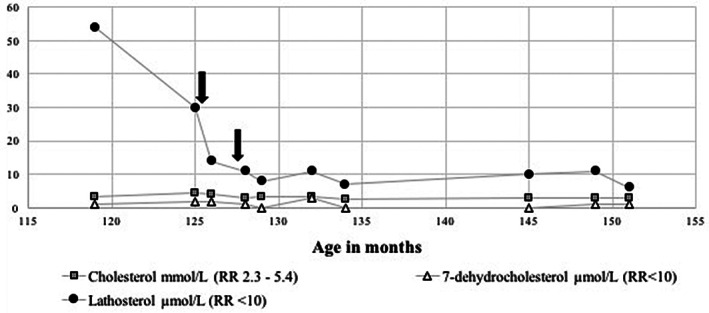
Plasma sterol levels before and after initiation of simvastatin therapy—5 mg daily (0.2 mg/kg/day) then 10 mg daily (0.4 mg/kg/day) (indicated by arrows)

The patient was treated with the cholesterol‐lowering agent simvastatin, 5 mg (0.2 mg/kg/day) initially, and the dose was increased to 10 mg daily (0.4 mg/kg/day) 8 weeks later. Serum creatine kinase levels were regularly monitored and ranged from 82 to 139 IU/L (RR: 30‐150 U/L). Serial plasma sterol assays showed near normalization of lathosterol levels (Figure 2) and liver function tests showed reduction in ALT and GGT levels after 8 weeks of initiating simvastatin. There was reduction in liver fibrosis 2 years after commencing simvastatin as evidenced by fibroscan readings (median reading reduced from 15.6 to 12.8 kPa) and improvement in liver enzyme levels (ALT reduced from 364 to 69 IU/L, GGT reduced from 414 to 136 IU/L).

## 3 DISCUSSION

To date, there are only seven reported patients with lathosterolosis including our patient.^2^, ^3^, ^4^, ^5^, ^6^, ^7^ There is variability in the severity of clinical features even within families as demonstrated by the sibling pair, patients 1 and 2 (Table 1). A pattern of microcephaly, bilateral cataracts, dysmorphism, limb anomalies, global developmental delay/intellectual disability, and liver involvement could be recognized among patients (Table 1). These clinical features closely resemble Smith‐Lemli‐Opitz syndrome (SLOS) which is due to deficient Δ^7^‐dehydrocholesterol reductase so that some patients with lathosterolosis were initially thought to have SLOS.^2^, ^4^, ^5^

**TABLE 1:** Clinical, biochemical, molecular, and outcome of patients with lathosterolosis

	Patient 1^2^	Patient 2^3^	Patient 3^4^	Patient 4^5^	Patient 5^6^	Patient 6^7^	Our patient
Main clinical features							
Age at diagnosis	7 years	Postmortem	Postmortem	22 months	10 years	9 years	10 years
Microcephaly	Yes	Yes	Yes	Yes	Yes	Yes	Yes
Bilateral cataracts (age noted)	Yes (6 years old)	No	Yes (at birth)	Small dots (4 years)	Posterior (5 years)	Yes (8 years)	Yes (5 years)
Dysmorphism	Yes	n/a	Yes	Yes	Yes	Yes	Yes
Other anomalies	Bilobate gallbladder	Arnold Chiari malformation type II	Ambigous genitalia	Single umbilical artery	None	None	Horseshoe kidney
	Horshoe kidney T8 butterfly vertebra	Lumbosacral meningocoele					
Limb anomalies (postaxial hexadactyly and/or second‐fourth toe syndactyly)	Yes	Yes	Yes	Yes	Clinodactyly only	Yes	Yes
Liver	Intrahepatic cholestasis, portal fibrosis, progressed to liver failure with portal hypertension liver transplantation at 8 years of age	Atrophy of hepatocytic laminae extramedullary hematopoiesis	Abnormal liver tests hepatosplenomegaly cirrhosis	Normal liver tests mild parenchymal disease	Normal liver tests	Abnormal liver tests hepatosplenomegaly cryptogenic cirrhosis portal hypertension	Abnormal liver tests portal and lobular hepatitis, focal and bridging fibrosis
Neurologic hypotonia	Yes	n/a	n/a	Yes	Yes	Yes	No
Global delay/ID	Yes, severe	n/a	Yes, severe	Yes, mild	Yes, mild autistic features	Yes	Yes, moderate
Investigations							
Cholesterol	Normal	n/a	n/a	Normal	Normal	n/a	Normal
7‐Dehydrocholesterol	Not detected	n/a	n/a	Normal	Normal	n/a	Normal
Lathosterol plasma	n/a	n/a	n/a	81.6 μmol/L (N < 18)	219.8 μmol/L (N < 16)	31.4 mg/L (N < 3.0)	54 μmol/L (N < 10)
Fibroblasts (% of total sterols)	12.5% after 15 days	n/a	35% after 6 days	1.48% after 10 days	n/a	n/a	n/a
8(9)‐Cholestanol (% of total sterols)	Not detected to trace	n/a	n/a	17.53%	21.61 μmol/L (N < 4)	n/a	Elevated
Molecular findings	c.88G>A; p.(R29Q)	c.88G>A; p. (R29Q)	c.137 A>C; p.(Y46S)	c.442A>G; p.(K148E)	c.479C>G; p.(P160R)	c.632G>A; p.(G211D)	c.212A>T; p.(N71I)
	c.632G>A; p.(G211D)	c.632G>A; p.(G211D)	c.137A>C; p.(Y46S)	c.630C>A; p.(D210E)	c.630C>A; p.(D210E)	c.632G>A; p.(G211D)	c.214C>T; p.(Q72*)
Simvastatin treatment	n/a	n/a	n/a	Yes	Yes	Yes	Yes
Dose				0.2 mg/kg/day up to 1 mg/kg/day	40 mg	Initial 10 mg then 20 mg	Initial—0.2 mg/kg/day then 0.4 mg/kg/day
Duration of treatment	n/a	n/a	n/a	3 years	4 months	4 months	2 years
Outcome	Alive at 7 years old	Aborted at 21 weeks	Died at 18 weeks	Alive at 5 years old	Alive at 11 years old	Alive at 9 years old	Alive at 13 years old

Abbreviations: ID, intellectual disability; n/a, not available.

The most common dysmorphic features in patients with lathosterolosis were epicanthic folds, receding forehead, bitemporal narrowing, micrognathia, high‐arched palate, broad nasal tip, anteverted nares, and ptosis. Postaxial hexadactyly and second‐fourth toe syndactyly were the most common observed limb anomalies. Skeletal abnormalities, fracture, osteoporosis, and vitamin D deficiency were reported.^2^, ^6^ Although our patient had normal 25OH vitamin D levels and bone mineral density was within the lower 25% of normal with height adjustment, it is important to continue monitoring these parameters due to a potential risk of low vitamin D secondary to deficient 7‐dehydrocholesterol, a vitamin D sterol precursor. Neurologic features included hypotonia (4/7) and mild to severe psychomotor retardation/intellectual disability in all surviving patients. Liver involvement was likewise variable as summarized in Table 1.

There are several proposed mechanisms underlying the malformations and phenotypic differences in patients with defects in the cholesterol biosynthesis pathway. They include the effects of cholesterol or sterol deficiency, specific teratogenic effects of the bioactive cholesterol precursors, and direct toxic effects.^4^, ^7^ The accumulating sterol intermediates due to their structural differences could substitute for cholesterol functionally and influence phenotypic expression or penetrance.^4^ In addition, the accumulation of lathosterol is hypothesized to be hepatotoxic and to cause cholestasis due to aberrant bile acid synthesis.^7^

Diagnosis of lathosterolosis was made in our patient by whole exome sequencing, but this could have been done by a complete sterol analysis. However, some laboratories may only measure 7‐dehydrocholesterol (7DHC) and 8‐dehydrocholesterol (8DHC). It is therefore important to be aware of the limitations of the sterol analysis offered in each laboratory so that a complete sterol analysis can be pursued especially when there is a clinical suspicion of a defect in cholesterol biosynthesis and a normal 7DHC.

Simvastatin is an inhibitor of HMG‐CoA reductase which converts HMG‐CoA to mevalonate, an early but rate limiting step in cholesterol synthesis. It has an advantage over other statins because of its lipophilic properties allowing it to cross the blood brain barrier and potentially inhibits cerebral cholesterol precursor accumulation.^8^ Simvastatin has been shown to decrease 7DHC and paradoxically increase cholesterol levels in plasma and cerebrospinal fluid of SLOS patients.^8^ The cholesterol biosynthetic pathway is mediated by a negative control feedback mechanism involving the Steroid Responsive Element Binding Proteins (SREBPs), that is, SREBP2 which are transcriptional factors regulating the enzymes involved in the pathway.^9^ The significant block of HMG‐CoA reductase using simvastatin results in the increase of all enzymes in the cholesterol biosynthesis pathway via the SREBPs and lowers accumulating sterols such as 7DHC and lathosterol. Studies using SLOS fibroblast cell lines have supported the beneficial effects of simvastatin only in those with residual enzyme activity.^10^

Simvastatin was given to three lathosterolosis patients to reduce lathosterol levels using the same rationale as its use in SLOS patients.^5^, ^6^, ^7^ Aside from significant lowering of lathosterol levels in these patients, one of the patient also had a mild improvement in developmental quotient.^5^

Liver transplantation (LT) was performed in one patient who had intrahepatic cholestasis and fibrosis progressing to liver failure with portal hypertension and this led to complete biochemical recovery and halted progression of neurological damage.^2^, ^3^, ^11^ As our patient had significantly abnormal liver function tests, we thought that treatment was worthwhile with the aim of preventing progressive fibrosis/cirrhosis necessitating liver transplant. Based on a previous case report,^5^ we used simvastatin initially at a dose of 5 mg daily (0.2 mg/kg/day). As there was no significant change in liver function tests, the dose was cautiously increased to 10 mg daily (0.4 mg/kg/day) 8 weeks later. Plasma sterol analysis demonstrated reduction in lathosterol levels (Figure 2). Simvastatin therapy in our patient not only led to decrease in serum ALT and GGT levels, but there was an associated improvement of liver fibrosis grades as evidenced by fibroscan. It is unclear whether prolonged treatment beyond 2 years in our patient will lead to a further reduction in fibrosis score.

## 4 CONCLUSION

This report describes a rare cause of abnormal liver function tests in association with dysmorphic features, cataracts, limb anomalies, and developmental delay/intellectual disability. Simvastatin treatment resulted in lowering of plasma lathosterol levels, improvement in liver function tests and liver fibrosis grade, suggesting that children with this condition should be actively treated in order to prevent progression of liver disease. Furthermore, our case illustrates the importance of including additional sterols such as lathosterol in sterol analysis particularly if the clinical features strongly suggest a disorder in cholesterol biosynthetic pathway.

## CONFLICT OF INTEREST

Joy Yaplito‐Lee, Gautham Pai, Winita Hardikar, Kai M. Hong, James Pitt, Justine Marum, and David J. Amor declare that they have no conflict of interest.

## AUTHOR CONTRIBUTIONS

Joy Yaplito‐Lee, Gautham Pai, David J. Amor, and Winita Hardikar were involved in clinical management of the patient, collecting the data, and drafting the manuscript. David J. Amor, Kai M. Hong, James Pitt, and Justine Marum were involved with the laboratory and genetic studies, collecting the data, and provided intellectual inputs in drafting the manuscript. All authors have inspected and approved the final draft of the manuscript. Joy Yaplito‐Lee will serve as the guarantor.

## INFORMED CONSENT

All procedures followed were in accordance with the ethical standards of the responsible committee on human experimentation (institutional and national) and with the Helsinki Declaration of 1975, as revised in 2000 (5). Informed consent was obtained from the patient for being included in the case report.

Additional informed consent was obtained from the patient for which identifying information was included in this article.

## ANIMAL RIGHTS

This article does not contain any studies with human or animal subjects performed by the any of the authors.

## GUARANTOR OF THE ARTICLE

Joy Yaplito‐Lee.

## ETHICS APPROVAL

Not required.
